# Mid to Long-Term Outcomes of Low-Energy Selective Laser Trabeculoplasty for Primary Open Angle Glaucoma

**DOI:** 10.3390/jcm15176631

**Published:** 2026-08-27

**Authors:** Hinako Ohtani, Yuri Fujino, Keigo Takagi, Yuto Yoshida, Chisako Ida, Kana Murakami, Mizuki Koike, Masaki Tanito

**Affiliations:** Department of Ophthalmology, Shimane University Faculty of Medicine, Izumo 693-8501, Japan

**Keywords:** selective laser trabeculoplasty (SLT), low energy, intraocular pressure (IOP), outcome, glaucoma

## Abstract

**Background/Objectives**: To evaluate the mid- to long-term outcomes of selective laser trabeculoplasty (SLT) performed with lower energy (≤0.5 millijoules (mJ)) than conventional settings (0.6–1.2 mJ). **Methods**: This retrospective chart-review study included patients who underwent low-energy SLT (0.34 ± 0.06 mJ) at Shimane University Hospital from April 2018 onward. A total of 54 patients (99 eyes) were analyzed, with a mean follow-up period of 57.5 months and a maximum follow-up period of 114 months. Treatment failure was defined using two criteria: an increase in the number of medications or the addition of incisional glaucoma surgery, and the addition of incisional glaucoma surgery. Kaplan–Meier survival analyses and multivariate Cox proportional hazards models were performed to evaluate long-term outcomes and prognostic factors. **Results**: During the follow-up period, 54 eyes underwent a single SLT treatment. A total of 37 eyes underwent two SLT treatments, and 8 eyes underwent three treatments. Mean intraocular pressure (IOP) significantly decreased from 16.7 mmHg at baseline to 15.1, 14.4, and 13.9 mmHg at 12, 36, and 60 months, respectively (mixed-effects model, *p* < 0.0001). Medication scores were 2.2, 2.3, 2.1, and 2.8 at baseline, 12, 36, and 60 months, respectively (*p* = 0.0006). Success rates at 12, 36, and 60 months were 72%, 37%, and 26% by Definition 1, and 90%, 69%, and 61% by Definition 2. At 36 months, success rates stratified by baseline medication score (0, 1–2, and ≥3) were 11%, 50%, and 38% for Definition 1 (*p* = 0.0002), and 83%, 75%, and 58% for Definition 2 (*p* = 0.02). Under Definition 1, more than 90% of eyes in the medication-free group required additional medications or surgery within 5 years. Under Definition 2, 60% of eyes with a preoperative medication score of ≥3 required surgeries within 5 years. **Conclusions**: Performing low-energy SLT in patients using approximately one medication can delay the need for additional therapy or surgery while maintaining IOP reduction over the observation period.

## 1. Introduction

Selective laser trabeculoplasty (SLT) is a laser treatment that selectively targets pigmented trabecular meshwork cells, thereby enhancing aqueous humor outflow through the conventional pathway [1]. Because SLT induces minimal thermal damage to the trabecular meshwork and preserves tissue architecture [2,3], it is considered a minimally invasive and repeatable procedure. The Laser in Glaucoma and Ocular Hypertension (LiGHT) trial represents a landmark study that established SLT as a first-line treatment for POAG and ocular hypertension [4,5]. This multicenter randomized controlled trial demonstrated that initial SLT achieved IOP control comparable to or better than topical medical therapy, while reducing the need for medications and improving cost-effectiveness over long-term follow-up. Notably, a substantial proportion of patients treated with SLT remained medication-free for several years, supporting the role of SLT as a durable and minimally invasive therapeutic option. Based on these findings, the LiGHT trial has significantly influenced current clinical practice by positioning SLT as an effective alternative to pharmacological therapy in the initial management of glaucoma.

Conventional SLT is typically performed at an energy level of approximately 0.6 to 1.2 millijoules (mJ), with the laser energy adjusted until so-called “champagne bubbles” are observed at the trabecular meshwork [6]. This strategy has been used to balance treatment efficacy with procedural safety and has become standard practice in many clinical settings [6,7]. In recent years, there has been growing interest in optimizing SLT laser parameters to minimize treatment-related inflammation, discomfort, and transient IOP spikes while maintaining pressure-lowering efficacy. Several studies have reported that low-energy SLT protocols may achieve comparable short-term IOP reduction with fewer adverse effects [8]. Notably, the Clarifying the Optimal Application of SLT Treatment (COAST) trial systematically evaluated different SLT energy and treatment protocols and demonstrated that low-energy approaches could provide effective IOP reduction while potentially reducing postoperative inflammation and patient discomfort [9,10,11]. Despite these promising findings, most previous studies have focused primarily on short-term outcomes, typically within 12 to 24 months after treatment. Consequently, data on the mid- to long-term efficacy and durability of low-energy SLT remain limited. There is a lack of evidence regarding long-term treatment survival, the need for additional medications or surgery, and prognostic factors influencing outcomes after low-energy SLT. These gaps in knowledge are clinically relevant, given the increasing emphasis on minimally invasive glaucoma therapies and individualized treatment strategies. Therefore, further investigation of the mid- to long-term outcomes of low-energy SLT is warranted to clarify its durability, identify patient subgroups most likely to benefit, and determine its optimal role within contemporary glaucoma management. In our institution, low-energy SLT, typically performed at 0.4 mJ without using the appearance of “champagne bubbles” as the treatment endpoint, has been routinely adopted since 2018. In the present study, we retrospectively investigated the mid- to long-term clinical outcomes of low-energy SLT in patients with glaucoma.

## 2. Subjects and Methods

### 2.1. Participants

This investigation was conducted in accordance with the Declaration of Helsinki and was approved by the Shimane University Hospital institutional review board (KS20200517-1; latest approval, 28 January 2026). Written consent for the SLT procedure was obtained before treatment. For this retrospective analysis, the IRB waived the requirement for separate written consent for publication; information about the study was instead made publicly available at the participating institution. All analyses used de-identified clinical data.

We retrospectively reviewed patients who received SLT at Shimane University Hospital from April 2018 onward. The inclusion criteria were a diagnosis of POAG, treatment with low-energy SLT (≤0.5 mJ), and a minimum follow-up period of 30 months. The exclusion criteria were glaucoma other than POAG, treatment with SLT at an energy level of >0.5 mJ, and a history of incisional glaucoma surgery or vitreoretinal surgery. A history of uncomplicated cataract surgery was not considered an exclusion criterion. Among treatment-naïve eyes, some patients had been referred from local ophthalmology clinics for further evaluation and confirmation of the diagnosis of glaucoma. Following comprehensive assessment at our institution, low-energy SLT was selected as the initial treatment without prior medical therapy. Repeat SLT treatments were permitted during follow-up when clinically indicated. As a result, 54 patients (99 eyes) met the eligibility criteria and underwent a total of 152 SLT sessions during the study period. The mean follow-up duration was 57.5 months, with a maximum follow-up duration of 114 months. The study parameters were evaluated at baseline and at 6-month intervals after treatment (6, 12, 18, 24, 30, 36, 42, 48, 54, 60, 66, 72, 78, 84, 90, 96, 102, 108, and 114 months), with an allowable window of ±2 months. The following preoperative data were collected through a medical chart review: age, sex, eye laterality, axial length measured using the OA-2000 Optical Biometer (Tomey, Nagoya, Japan), the Shimane University prostaglandin-associated periorbitopathy grading system (SU-PAP) score [12], and a history of cataract surgery. Intraoperative data included SLT energy and the number of laser applications. Parameters evaluated both before and after SLT included IOP measured by Goldmann applanation tonometry, the number of antiglaucoma medications, best-corrected visual acuity (BCVA), corneal endothelial cell density (CECD) measured using the EM-3000 specular microscope (Tomey, Nagoya, Japan), anterior chamber (AC) flare measured using the FM-600 laser flare meter (Kowa, Nagoya, Japan), visual field mean deviation (MD) assessed using the Central 30-2 Program of the Humphrey Visual Field Analyzer (Carl Zeiss Meditec AG, Oberkochen, Germany), the number of repeat SLT treatments, the need for additional glaucoma medications, subsequent cataract surgery, subsequent incisional glaucoma surgery, and post-treatment complications. At our institution, CECD, AC flare, and visual field examinations are routinely performed during follow-up as part of the standard clinical evaluation to monitor the long-term safety and efficacy of glaucoma treatment. In addition, repeat SLT treatments were performed in patients who required further IOP control during follow-up. Missing data were imputed using the last observation carried forward (LOCF) method. Treatment failure was evaluated according to two criteria. Under Definition 1, failure was defined as an increase in the number of antiglaucoma medications or the addition of incisional glaucoma surgery. Under Definition 2, failure was defined as the addition of incisional glaucoma surgery alone. Therefore, eyes that underwent glaucoma surgery after prior medication escalation were counted only under Definition 2 and not as surgical failures under Definition 1.

### 2.2. Low-Energy SLT Procedures

Low-energy SLT was performed using either the Tango Neo laser system (Ellex, Adelaide, Australia) or the YC-200 S Plus laser system (NIDEK, Gamagori, Japan). A Latina SLT Gonio Lens (Ocular Instruments, Bellevue, WA, USA) or a Rapid SLT Lens (Volk Optical Inc., Mentor, OH, USA) was used for laser delivery. The procedures were performed by 13 treating physicians. Prior to treatment, one drop each of 1% pilocarpine hydrochloride ophthalmic solution (Sanpilo; Santen Pharmaceutical, Osaka, Japan), oxybuprocaine hydrochloride ophthalmic solution (Benoxil; Santen Pharmaceutical, Osaka, Japan), and apraclonidine hydrochloride ophthalmic solution (Iopidine; Alcon, Geneva, Switzerland) was instilled. SLT was performed over 360° of the trabecular meshwork using a laser energy of ≤0.5 mJ. Laser spots were applied in a non-overlapping manner throughout the treatment area. One drop of apraclonidine hydrochloride ophthalmic solution was instilled immediately after the procedure. No postoperative topical corticosteroids or nonsteroidal anti-inflammatory drugs (NSAIDs) were administered. Preexisting glaucoma medications were continued after SLT.

### 2.3. Statistical Analysis

Longitudinal IOP and medication data were analyzed with mixed-effects regression to account for correlated observations from fellow eyes of the same patient. Patient identifier was entered as a random effect, whereas follow-up time and glaucoma surgical procedure were modeled as fixed effects. Individual postoperative time points were also compared with baseline using the Wilcoxon signed-rank test.

We analyzed treatment survival using Kaplan–Meier survival curves. Treatment failure was evaluated according to two criteria. Under Definition 1, failure was defined as an increase in the number of antiglaucoma medications or the addition of incisional glaucoma surgery. Under Definition 2, failure was defined as the addition of incisional glaucoma surgery alone. Eyes meeting either failure criterion were considered uncensored events in the survival analysis. Repeat SLT was not considered a treatment failure. The LiGHT study demonstrated that repeat SLT effectively maintained IOP control in eyes requiring retreatment [5], while the COAST trial was specifically designed on the premise that repeated low-energy SLT may prolong medication-free disease control [9]. Therefore, repeat SLT was regarded as part of the treatment strategy rather than a failure event in the present study. Survival distributions were compared across baseline medication groups (0, 1–2, or ≥3 components) with log-rank tests. To identify risk factors for failure, multivariable analysis was performed using a Cox proportional hazards model, and *p*-values were calculated using the Wald test.

All tests were two-tailed with statistical significance set at *p* < 0.05. Continuous data are reported as mean ± standard deviation (SD), whereas categorical data are given as counts and percentages. Decimal visual acuity was transformed to logMAR before analysis. Statistical calculations were performed in JMP Pro 14.2 (SAS Institute Inc., Cary, NC, USA). ChatGPT 5.5 (OpenAI, San Francisco, CA, USA) was used solely for English language editing and to improve the clarity and readability of the manuscript. ChatGPT was not used for study design, data collection, data analysis, interpretation of the results, or generation of scientific conclusions. All AI-assisted content was carefully reviewed and verified by the authors, who take full responsibility for the final content of the manuscript.

## 3. Results

Baseline demographic and clinical characteristics are summarized in Table 1. The mean baseline IOP was 16.7 ± 3.8 mmHg, and the mean number of medications, expressed as one point per active antiglaucoma medication ingredient, was 2.2 ± 1.3. At baseline, 18 eyes were medication-free, 36 eyes were receiving one to two medication ingredients, and 45 eyes were receiving three or more medication ingredients. Preoperative BCVA was −0.04 ± 0.11 logMAR. The mean Humphrey visual field MD was −7.8 ± 6.3 decibel (dB). The mean SU-PAP score was 0.75 ± 0.67. Previous cataract surgery had been performed in 12.1% of eyes.

SLT treatment parameters and follow-up data are shown in Table 2. During the follow-up period, a total of 152 SLT sessions were performed. Of the 99 eyes, 54 underwent a single SLT treatment, 37 underwent two SLT treatments, and 8 underwent three SLT treatments. Among the 45 eyes that underwent repeat SLT, the mean interval between the first and second SLT treatments was 31.3 ± 12.8 months. Among the eight eyes that underwent a third SLT treatment, the mean interval between the first and third SLT treatments was 64.5 ± 20.0 months. The mean laser energy per treatment was 0.34 ± 0.06 mJ, and the mean number of laser applications was 71.1 ± 7.1 shots. All SLT procedures were performed using a laser energy of ≤0.5 mJ. No SLT-related complications were observed during the study period.

Changes in IOP and the number of medications after low-energy SLT are summarized in Table 3 and Table 4, respectively. Mixed-effects models demonstrated significant changes in IOP (Table 3, *p* < 0.0001) and in the number of medications (Table 4, *p* = 0.0006). IOP showed a significant reduction from 1 to 4 years after SLT, decreasing by 1.6–3.4 mmHg from a baseline of 16.7 mmHg, which corresponds to a 9.5–20.4% reduction. The number of medications remained largely unchanged; however, it increased to 2.3 at 1 year and 2.8 at 5 years. These findings indicate that SLT, even at lower energy settings, reduced IOP, while the medication burden remained unchanged or increased.

Changes in BCVA and Humphrey visual field MD after low-energy SLT are summarized in Table 5 and Table 6, respectively. Mixed-effects models demonstrated no significant longitudinal changes in BCVA (Table 5, *p* = 0.64) or MD (Table 6, *p* = 0.30). The mean preoperative BCVA was −0.04 ± 0.1 logMAR and remained stable throughout the follow-up period, ranging from −0.05 to −0.02 logMAR. Although a statistically significant difference from baseline was observed at 2 years (*p* = 0.03), no significant differences were detected at the other time points. The mean preoperative MD was −7.8 ± 6.3 dB. MD values ranged from −8.1 to −8.8 dB during follow-up. While MD was significantly lower than baseline at 2 years and thereafter according to pairwise comparisons, the mixed-effects model showed no significant overall longitudinal change. These findings suggest that visual acuity and visual field status were generally maintained during long-term follow-up after low-energy SLT.

Changes in CECD and AC flare after low-energy SLT are summarized in Table 7 and Table 8, respectively. Mixed-effects models demonstrated significant longitudinal changes in both CECD (Table 7, *p* < 0.0001) and anterior chamber flare (Table 8, *p* = 0.04). The mean preoperative CECD was 2640 ± 312 cells/mm^2^ and gradually decreased throughout the follow-up period, reaching 2446 ± 396 cells/mm^2^ at 5 years. Compared with baseline, CECD was significantly reduced at all postoperative time points. The mean preoperative anterior chamber flare was 7.2 ± 4.9 pc/ms. Flare values showed a gradual increase over time, reaching 9.8 ± 8.3 pc/ms at 5 years. Compared with baseline, flare measurements were significantly higher from 2 years onward. These findings indicate that low-energy SLT was associated with a gradual decrease in corneal endothelial cell density and a mild increase in anterior chamber flare during long-term follow-up.

**Figure 1:**
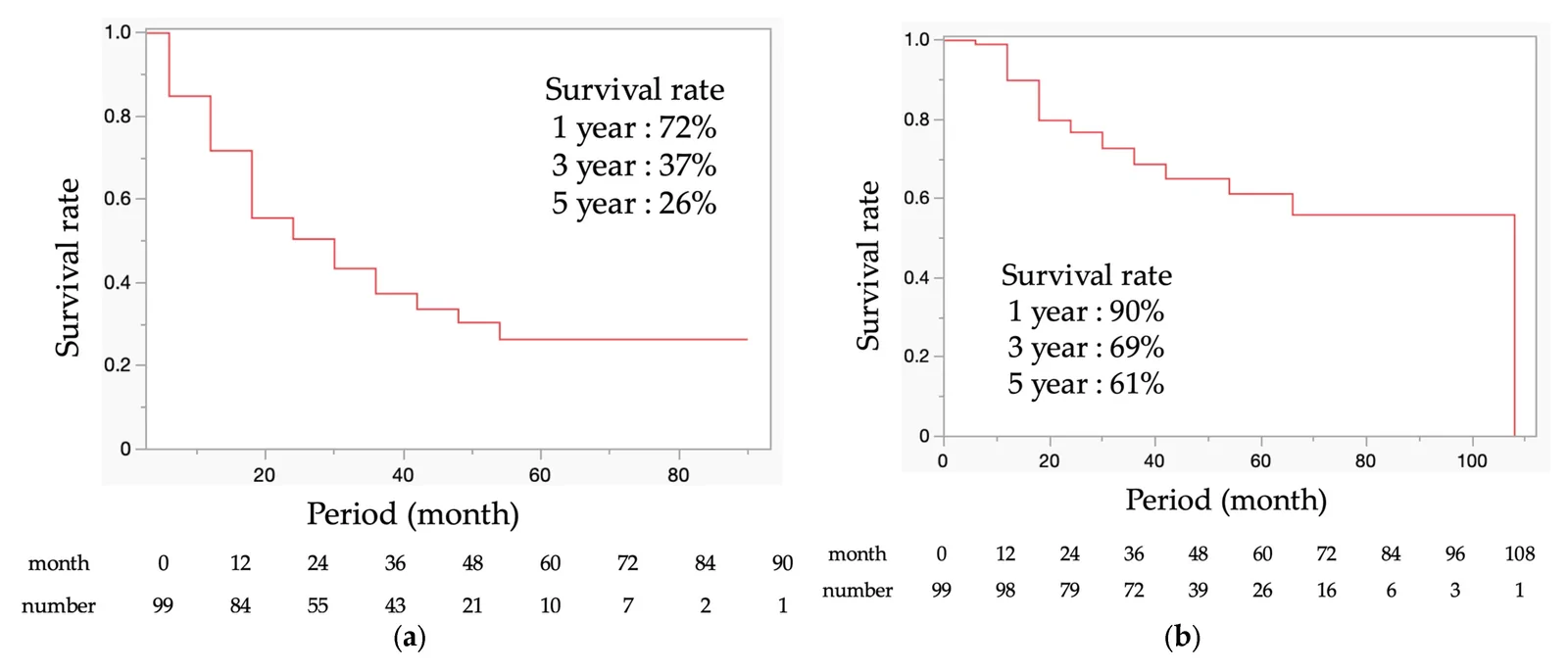
Kaplan–Meier survival curves following low-energy SLT. Two failure criteria were applied: Definition 1 (**a**), defined as an increase in medication score and/or the addition of incisional glaucoma surgery; and Definition 2 (**b**), defined as the addition of incisional glaucoma surgery.

Kaplan–Meier survival curves under Definitions 1 and 2 are shown in Figure 1. Under Definition 1 (failure defined as an increase in the number of medications or the addition of incisional glaucoma surgery), the survival rates without additional eye drops or incisional glaucoma surgery after SLT were 72% at 1 year, 37% at 3 years, and 26% at 5 years (Figure 1a). Under Definition 2 (failure defined as the addition of incisional glaucoma surgery only), the survival rates were 90% at 1 year, 69% at 3 years, and 61% at 5 years (Figure 1b).

The causes of failure under Definitions 1 and 2 are summarized in Table 9. Under Definition 1, treatment failure was defined as the first occurrence of either an increase in antiglaucoma medications or the addition of incisional glaucoma surgery. Accordingly, 45 eyes failed because of medication escalation and 27 eyes failed because of incisional glaucoma surgery. The surgical procedures listed under Definition 1 correspond to these 27 eyes, whereas those listed under Definition 2 correspond to all 40 eyes that underwent incisional glaucoma surgery during follow-up. Among the additional incisional glaucoma surgeries, 70% in Definition 1 and 67% in Definition 2 were angle surgeries including trabeculotomy (LOT) or iStent implantation, indicating that most additional surgeries were minimally invasive glaucoma surgeries (MIGS). No eyes underwent standalone cataract surgery during follow-up; all cataract surgeries were combined with MIGS.

Multivariate analyses using a Cox proportional hazards model were conducted to identify factors associated with failure under Definitions 1 and 2. The results of the Cox proportional hazards models are presented in Table 10. Under Definition 1, higher baseline IOP and preoperative medication score were significant risk factors for failure (failure defined as additional eye drops or incisional glaucoma surgery). Under Definition 2, older age, higher baseline IOP, and worse visual field MD and preoperative medication score were significant risk factors for failure (failure defined as additional incisional glaucoma surgery).

**Figure 2:**
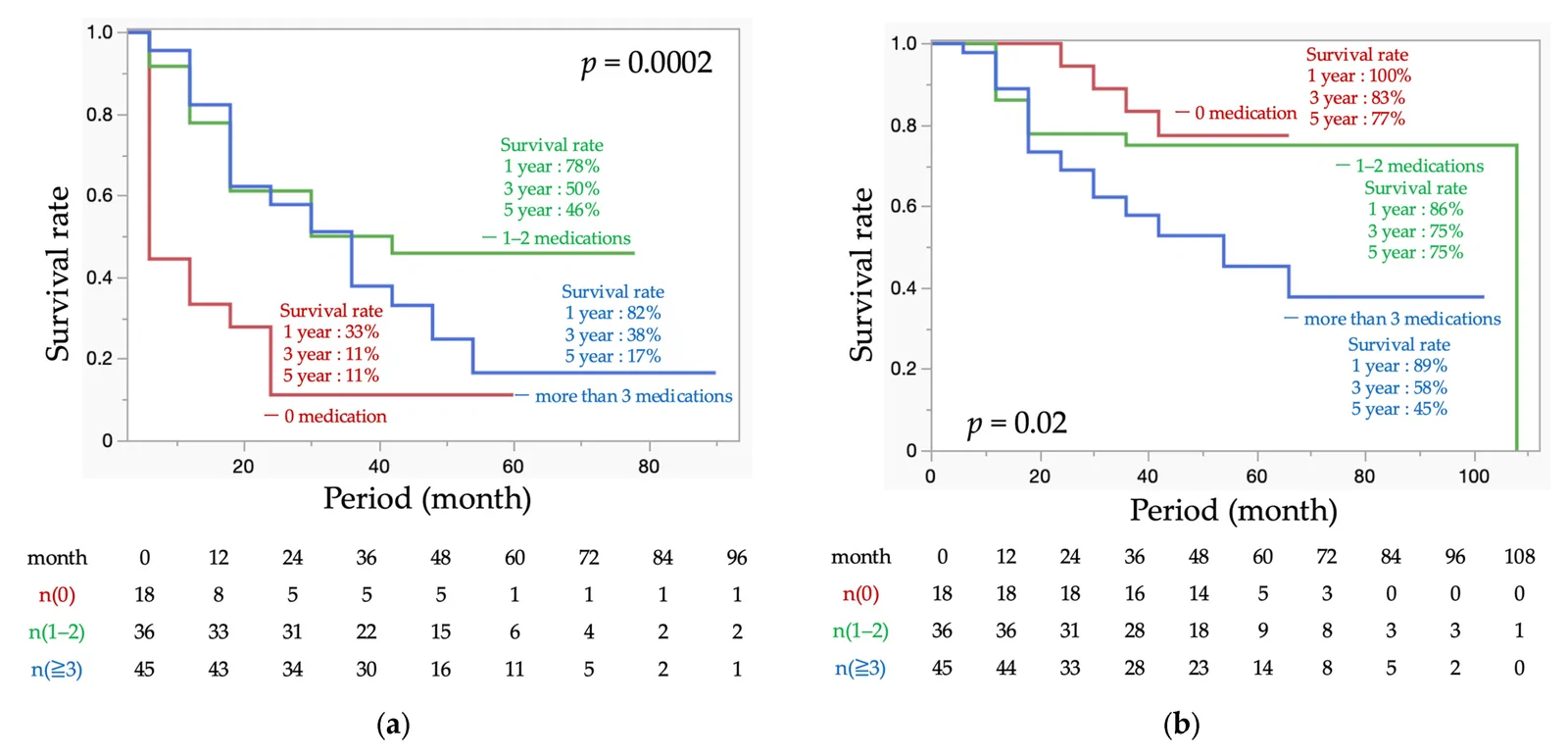
Kaplan–Meier survival curves following low-energy SLT stratified by preoperative medication score. Two failure criteria were applied: Definition 1 (**a**), defined as an increase in medication score and/or the addition of incisional glaucoma surgery; and Definition 2 (**b**), defined as the addition of incisional glaucoma surgery. Patients were stratified into three groups according to the preoperative number of antiglaucoma medications (0, 1–2, and ≥3). Survival distributions were compared using the log-rank test.

Because high IOP and preoperative medication score were significant risk factors for both criteria, we performed Kaplan–Meier survival analyses stratified by preoperative medication score. Under Definition 1, eyes were classified into three groups: 0, 1–2, and ≥3 medications (Figure 2a). In the 0-medication group treated with SLT without prior therapy, the survival rates were 33% at 1 year, 11% at 3 years, and 11% at 5 years. In the 1–2-medication group, the survival rates were 78% at 1 year, 50% at 3 years, and 46% at 5 years. In the ≥3-medication group, the survival rates were 82% at 1 year, 38% at 3 years, and 17% at 5 years. Survival differed significantly among the three groups (log-rank test, *p* = 0.0002). Under Definition 2 (Figure 2b), in the 0-medication group, the survival rates were 100% at 1 year, 83% at 3 years, and 77% at 5 years. In the 1–2 medication group, the survival rates were 86% at 1 year, 75% at 3 years, and 75% at 5 years. In the ≥3-medication group, the survival rates were 89% at 1 year, 58% at 3 years, and 45% at 5 years. Survival also differed significantly among the three groups (log-rank test, *p* = 0.02).

Under Definition 1, more than 90% of eyes in the medication-free group required additional medications or surgery within 5 years, suggesting that achieving medication-free control with SLT may be difficult. Under Definition 2, 60% of eyes with a preoperative medication score of ≥3 required surgery within 5 years, suggesting that SLT may be less effective in avoiding surgery in advanced cases.

## 4. Discussion

In this study, low-energy SLT (≤0.5 mJ) achieved an overall longitudinal reduction in IOP over a mean follow-up period of 57.5 months while maintaining visual acuity and visual field status. Although the number of antiglaucoma medications remained largely unchanged throughout follow-up, survival analyses demonstrated that treatment outcomes differed substantially depending on the definition of failure and the preoperative medication burden. Under the definition that included medication escalation, approximately three-quarters of eyes required additional treatment within 5 years. In contrast, when failure was defined as the need for incisional glaucoma surgery alone, more than half of eyes remained surgery-free at 5 years. Multivariable Cox analyses further identified higher baseline IOP and greater preoperative medication burden as independent predictors of treatment failure, whereas older age and more advanced visual field loss were additional risk factors for subsequent glaucoma surgery. Importantly, stratified Kaplan–Meier analyses demonstrated that eyes receiving one to two medications before SLT achieved the most favorable long-term outcomes, whereas treatment-naïve eyes frequently required subsequent medications and eyes receiving three or more medications often progressed to surgery. These findings suggest that the long-term value of low-energy SLT depends not only on its IOP-lowering efficacy but also on appropriate patient selection.

Previous studies have consistently shown that low-energy SLT achieves short-term IOP reductions comparable to those of conventional SLT despite the use of substantially lower laser energy. Tang et al., Xu et al., and more recently Sacks et al. reported IOP reductions ranging from approximately 2 to 5 mmHg, with percentage reductions of approximately 10–25% [8,13,14]. Our findings are consistent with these reports despite a relatively low baseline IOP of 16.7 mmHg. Although the absolute IOP reduction in the present study was modest (1.6–3.4 mmHg), the percentage reduction (9.5–20.4%) was comparable to previous studies. This observation is consistent with the well-recognized relationship between baseline IOP and the magnitude of pressure reduction after SLT.

A major strength of the present study is the evaluation of long-term treatment durability according to preoperative medication burden. Previous studies have primarily focused on short-term IOP reduction, whereas few have investigated which patients derive the greatest long-term benefit from low-energy SLT [8,13,14]. Our Kaplan–Meier analyses demonstrated distinct clinical patterns among medication groups. In treatment-naïve eyes, more than 90% eventually required additional medication or surgery, suggesting that low-energy SLT alone is unlikely to maintain long-term medication-free disease control. Conversely, among eyes receiving three or more medications, approximately 60% underwent incisional glaucoma surgery within 5 years, indicating that low-energy SLT may be insufficient to delay surgical intervention in more advanced disease. In contrast, patients receiving one to two medications experienced the highest long-term treatment survival under both failure definitions. These findings suggest that, in the context of maintaining the current medical treatment regimen, low-energy SLT may be most appropriately positioned as an early adjunctive treatment rather than as primary therapy or a rescue treatment for advanced glaucoma.

In this study, repeat SLT was not regarded as treatment failure because retreatment has become an accepted component of contemporary SLT management. This approach is supported by both the LiGHT study and the COAST trial, which demonstrated that repeat SLT can safely maintain IOP control and prolong disease control without immediate escalation to medications or surgery [4,5,9,10,11]. During follow-up, 45% of eyes underwent repeat SLT, indicating that retreatment played an important role in maintaining long-term pressure control in routine clinical practice. Interestingly, most eyes requiring additional surgery underwent minimally invasive glaucoma surgery, particularly trabeculotomy or iStent implantation, rather than filtration surgery. This finding suggests that low-energy SLT may successfully postpone the need for more invasive procedures, allowing many patients to undergo angle-based surgery as the initial surgical intervention. Whether low-energy SLT contributes to delaying progression to filtration surgery warrants further investigation.

A unique strength of the present study is the availability of comprehensive longitudinal safety data. At our institution, BCVA, IOP, Humphrey visual field testing, CECD, and AC flare are routinely assessed during follow-up as part of standard glaucoma care. This practice enabled a comprehensive evaluation of both the long-term efficacy and safety of low-energy SLT. Although conventional SLT is generally considered a safe procedure, transient complications such as IOP spikes, anterior chamber inflammation, corneal edema, and corneal endothelial changes have been reported, and rare cases of permanent corneal complications have also been described [15]. In contrast, no clinically significant postoperative complications, including transient IOP spikes or anterior chamber inflammation, were observed after low-energy SLT in the present study, and both visual acuity and visual field remained stable throughout the follow-up period. Although CECD gradually decreased and AC flare increased over time, these longitudinal changes should be interpreted with caution. CECD is known to decline physiologically with aging by approximately 0.25–0.5% per year [16]. Although the reduction observed in the present study was greater than the expected physiological age-related decline, the longitudinal analyses included eyes that subsequently underwent surgeries during follow-up, both of which may have contributed to endothelial cell loss. Therefore, the observed reduction in CECD cannot be attributed solely to low-energy SLT. Likewise, the increase in AC flare may have also been influenced by subsequent intraocular surgeries during follow-up.

This study has several limitations. First, this was a retrospective study, and therefore is subject to inherent limitations, including potential selection bias and incomplete control of confounding factors. Second, treatment protocols, including SLT energy settings and postoperative management, were not fully standardized across all cases, as procedures were performed by multiple physicians. In this study, the mean number of laser applications was 71.1, which was lower than the approximately 100 applications commonly used in standardized 360° SLT protocols [17]. Future prospective studies with standardized numbers of laser applications and spot spacing are warranted to validate our findings. Third, although both eyes of some patients were included in the analysis and a mixed-effects model was used to account for intra-patient correlation, residual bias cannot be completely excluded. In addition, the absence of a direct comparison group treated with conventional-energy SLT limits the ability to draw definitive conclusions regarding the relative efficacy of low-energy SLT. Fourth, the effect of the interval between repeated SLT treatments on treatment outcomes was not evaluated in this study. Further studies are needed to clarify its impact. Finally, although the follow-up period was relatively long, variations in follow-up intervals and the retrospective nature of data collection may have influenced the assessment of long-term outcomes. In conclusion, low-energy SLT provides a clinically meaningful and sustained intraocular pressure-lowering effect; however, its long-term effectiveness is influenced by baseline disease severity and medication burden, underscoring the importance of appropriate patient selection and optimized treatment strategies.

## 5. Conclusions

In conclusion, low-energy SLT (≤0.5 mJ) achieved significant and durable IOP reduction with minimal adverse events over long-term follow-up. However, many eyes required subsequent initiation or escalation of medical therapy during follow-up. Long-term medication-free control was uncommon in treatment-naïve eyes, and avoidance of glaucoma surgery remained challenging in eyes requiring three or more medication components. In contrast, patients receiving one to two medication components at baseline experienced the greatest benefit, with prolonged treatment survival and delayed escalation to additional medications or surgery. These findings suggest that low-energy SLT may be most appropriately used as an early adjunctive intervention in POAG, before substantial disease progression necessitates intensive medical or surgical management.

## Figures and Tables

**Table 1:** Demographic data.

Parameters	Values
Number, subjects	54
Age, years	
Mean ± SD	55.3 ± 12.8
95% CI	51.8, 58.8
Gender	
Male, n (%)	26 (48.1)
Female, n (%)	28 (51.9)
Number, eyes	99
Side	
Right, n (%)	53 (53.5)
Left, n (%)	46 (46.5)
Preoperative BCVA, logMAR	
Mean ± SD	−0.04 ± 0.11
95% CI	−0.06, −0.02
Preoperative IOP, mmHg	
Mean ± SD	16.7 ± 3.8
95% CI	15.9, 17.4
Number of medications	
Mean ± SD	2.2 ± 1.3
95% CI	1.9, 2.4
0 medication, n (%)	18 (18.2)
1–2 medications, n (%)	36 (36.4)
≥3 medications, n (%)	45 (45.5)
CECD, cells/mm^2^	
Mean ± SD	2640 ± 312
95% CI	2576, 2704
AC flare, pc/ms	
Mean ± SD	7.2 ± 4.9
95% CI	6.2, 8.2
MD, dB	
Mean ± SD	−7.8 ± 6.3
95% CI	−6.6, −9.1
Axial length, mm	
Mean ± SD	25.8 ± 1.7
95% CI	25.4, 26.1
SU-PAP score	
Mean ± SD	0.75 ± 0.67
95% CI	0.61, 0.89
Previous cataract surgery	
Yes, n (%)	12 (12.1)

SD, standard deviation; CI, confidence interval; POAG, primary open-angle glaucoma; BCVA, best-corrected visual acuity; IOP, intraocular pressure; CECD, corneal endothelial cell density; AC, anterior chamber; pc, photon count; ms, millisecond; MD, mean deviation; dB, decibel; SU-PAP, Shimane University prostaglandin-associated periorbitopathy grading system.

**Table 2:** SLT treatment parameters.

Parameters	Values
Shot energy, mJ	
Mean ± SD	0.34 ± 0.06
95% CI	0.33, 0.35
Shot count, n	
Mean ± SD	71.1 ± 7.1
95% CI	69.5, 72.5
Total energy, mJ	
Mean ± SD	22.1 ± 8.9
95% CI	20.4, 23.9
Number of SLT sessions	
Mean ± SD	1.7 ± 0.5
95% CI	1.6, 1.8
Once, n (%)	54 (54.5)
Twice, n (%)	37 (37.3)
Three times, n (%)	8 (8.1)

SLT, selective laser trabeculoplasty; mJ, millijoules; SD, standard deviation; CI, confidence interval.

**Table 3:** Changes in IOP.

Parameters	Pre-SLT	1 Year	2 Years	3 Years	4 Years	5 Years
Number	99	99	99	88	52	35
IOP, mmHg						
Mean ± SD	16.7 ± 3.8	15.1 ± 3.8	14.6 ± 3.4	14.4 ± 4.0	13.3 ± 3.1	13.9 ± 3.4
95% CI	15.9, 17.4	14.3, 15.9	13.9, 15.3	13.5, 15.2	12.4, 14.1	12.7, 15.0
IOP reduction, mmHg		1.6	2.1	2.3	3.4	2.8
IOP reduction, %		9.5	12.6	13.8	20.4	16.8
*p* value		0.0007 **	<0.001 **	<0.001 **	<0.001 **	0.12

IOP, intraocular pressure; SD, standard deviation; CI, confidence interval; IOP reduction (%) = (IOP before SLT—IOP after SLT)/(IOP before SLT) × 100. *p* values are calculated using the Wilcoxon signed-rank test by comparing each postoperative time point with the pre-SLT value. ** indicates significance level at 1% (*p* < 0.01).

**Table 4:** Changes in number of medications.

Parameters	Pre-SLT	1 Year	2 Years	3 Years	4 Years	5 Years
Number	99	99	99	99	99	36
Number of medications						
Mean ± SD	2.2 ± 1.3	2.3 ± 1.1	2.2 ± 1.1	2.2 ± 1.1	2.2 ± 1.1	2.8 ± 1.0
95% CI	1.9, 2.4	2.1, 2.6	2.0, 2.4	2.0, 2.4	2.0, 2.4	2.4, 3.1
*p* value		0.02 *	0.63	0.35	0.66	0.05 *

SD, standard deviation; CI, confidence interval. *p* values are calculated using the Wilcoxon signed-rank test by comparing each postoperative time point with the pre-SLT value. * indicates significance level at 5% (*p* < 0.05).

**Table 5:** Changes in visual acuity.

Parameters	Pre-SLT	1 Year	2 Years	3 Years	4 Years	5 Years
Number	99	99	99	88	52	36
BCVA, logMAR						
Mean ± SD	−0.04 ± 0.1	−0.03 ± 0.1	−0.03 ± 0.1	−0.02 ± 0.2	−0.05 ± 0.1	−0.05 ± 0.05
95% CI	−0.06, −0.02	−0.06, −0.01	−0.05, −0.01	−0.06, 0.01	−0.07, −0.03	−0.07, −0.04
*p* value		0.24	0.03 *	0.10	0.24	0.06

BCVA, best-corrected visual acuity; SD, standard deviation; CI, confidence interval. *p* values are calculated using the Wilcoxon signed-rank test by comparing each postoperative time point with the pre-SLT value. * indicates significance level at 5% (*p* < 0.05).

**Table 6:** Changes in Humphrey visual field MD.

Parameters	Pre-SLT	1 Year	2 Years	3 Years	4 Years	5 Years
Number	99	99	99	88	52	36
MD						
Mean ± SD	−7.8 ± 6.3	−8.3 ± 6.5	−8.3 ± 6.1	−8.8 ± 6.5	−8.1 ± 5.9	−8.7 ± 5.6
95% CI	−9.1, −6.6	−9.6, −7.0	−9.5, −7.1	−10.2, −7.5	−9,8, −6.5	−10.6, −6.8
*p* value		0.07	0.006 **	0.002 **	0.007 **	0.008 **

MD, mean deviation; SD, standard deviation; CI, confidence interval. *p* values are calculated using the Wilcoxon signed-rank test by comparing each postoperative time point with the pre-SLT value. ** indicates significance level at 1% (*p* < 0.01).

**Table 7:** Changes in corneal endothelial cell density.

Parameters	Pre-SLT	1 Year	2 Years	3 Years	4 Years	5 Years
Number	95	97	97	86	52	36
CECD, cells/mm^2^						
Mean ± SD	2640 ± 312	2594 ± 309	2570 ± 343	2569 ± 340	2527 ± 355	2446 ± 396
95% CI	2576, 2703	2532, 2656	2501, 2639	2496, 2642	2428, 2626	2312, 2580
*p* value		0.04 *	0.01 *	0.0007 **	<0.0001 **	<0.0001 **

CECD, corneal endothelial cell density; SD, standard deviation; CI, confidence interval. *p* values are calculated using the Wilcoxon signed-rank test by comparing each postoperative time point with the pre-SLT value. * and ** indicate significance levels at 5% (*p* < 0.05) and 1% (*p* < 0.01).

**Table 8:** Changes in anterior chamber flare.

Parameters	Pre-SLT	1 Year	2 Years	3 Years	4 Years	5 Years
Number	97	97	97	86	52	36
AC flare, pc/ms						
Mean ± SD	7.2 ± 4.9	7.4 ± 3.6	8.0 ± 4.7	8.3 ± 5.0	8.0 ± 3.2	9.8 ± 8.3
95% CI	6.2, 8.2	6.7, 8.1	7.1, 9.0	7.3, 9.4	7.1, 8.8	6.9, 12.6
*p* value		0.09	0.009 **	0.0003 **	<0.0001 **	<0.0001 **

AC, anterior chamber; pc, photon count; ms, millisecond; SD, standard deviation; CI, confidence interval. *p* values are calculated using the Wilcoxon signed-rank test by comparing each postoperative time point with the pre-SLT value. ** indicates significance level at 1% (*p* < 0.01).

**Table 9:** Causes of SLT failure.

Parameters	Definition 1, n (%)	Definition 2, n (%)
Additional medication	45 (45.4)	
Additional glaucoma surgery	27 (27.3)	40 (40.4)
Ahmed Glaucoma Valve implantation	0 (0)	1 (1.0)
Trabeculectomy	4 (4.0)	4 (4.0)
PreserFlo MicroShunt implantation	4 (4.0)	8 (8.1)
Trabeculotomy	16 (16.1)	23 (23.2)
iStent implantation	3 (3.0)	4 (4.0)

**Table 10:** Risk factors for failure of SLT.

Parameters	Definition 1			Definition 2		
	HR	95% CI	*p* Value	HR	95% CI	*p* Value
Age, /year	1.01	0.98, 1.04	0.48	1.07	1.03, 1.11	0.0002 **
Gender, male/female	0.79	0.44, 1.39	0.41	1.29	0.64, 2.57	0.48
Previous cataract surgery, yes/no	2.37	0.85, 6.60	0.10	0.78	0.17, 3.55	0.75
Axial length, /mm	1.12	0.93, 1.34	0.22	1.10	0.87, 1.38	0.42
Preoperative IOP, /mmHg	1.13	1.03, 1.24	0.008 **	1.24	1.09, 1.41	0.0009 **
Preoperative MD, /dB	0.96	0.91, 1.01	0.10	0.93	0.88, 0.99	0.02 *
Preoperative medication score			0.01 *			0.003 **
0/1–2	2.83	1.19, 6.76	0.02 *	0.25	0.06, 1.00	0.05
0/3	3.56	1.54, 8.23	0.003 **	0.11	0.03, 0.42	0.001 **
1–2/3	1.26	0.65, 2.43	0.50	0.45	0.19, 1.06	0.07

HR, hazard ratio; CI, confidence interval; IOP, intraocular pressure; MD, mean deviation; dB, decibel. Multivariable analysis was performed using a Cox proportional hazards model, and *p* values are calculated using the Wald test. * and ** indicate significance levels at 5% (*p* < 0.05) and 1% (*p* < 0.01).

## Data Availability

Data is fully available upon reasonable request to corresponding author.
